# Axial pelvic tilt in direct anterior Total hip Arthroplasty using a traction table

**DOI:** 10.1186/s12891-020-03837-7

**Published:** 2020-12-03

**Authors:** A. Aichmair, M. Dominkus, J. G. Hofstaetter

**Affiliations:** 1 II. Department of Orthopaedic Surgery, Orthopaedic Hospital Vienna-Speising, Vienna, Austria; 2 Michael Ogon Laboratory for Orthopaedic Research, Orthopaedic Hospital Vienna-Speising, Vienna, Austria

**Keywords:** Total hip arthroplasty, Minimally invasive, Direct anterior approach, Cup misplacement, Traction table

## Abstract

**Background:**

Direct anterior approach total hip arthroplasty may be undertaken on a traction table, but the effects that patient positioning can have on axial pelvic tilt (aPT) are unknown. The aim of this study was to assess the degree of error from patient positioning on the traction table during anterior minimally-invasive surgery (AMIS) THA.

**Methods:**

Patients were included who underwent direct anterior THA via the AMIS technique at a single institution between 11/2018 and 03/2019. Axial pelvic tilt was measured (a) in the supine position on the operating table, and (b) after positioning on the traction table, by the same consultant surgeon in all cases.

**Results:**

In the above-mentioned study period, 50 patients (F: 32; M: 18) with an average age of 60.6 ± 13.6 (range: 26.5 to 88.3) years, and an average BMI of 27.2 ± 5.0 (range: 17.9 to 41.5) kg/m^2^ met the inclusion criteria. When measured in supine position, the average aPT was − 0.2 ± 1.7 (range: − 5.6 to 3.8) degrees. After positioning on the traction table, the average aPT was − 3.5 ± 2.1 (− 8.5 to 1.6) degrees (*p* < 0.001). In patients with an aPT of more than 5 degrees, the caput-collum-diaphyseal (CCD) angle was significantly lower (125 ± 11° vs. 134 ± 8°, *p* = 0.007).

**Conclusion:**

This study raises awareness for the potential risk of aPT during positioning of the patient on the traction table, commonly used during direct anterior THA via the AMIS technique.

## Background

Osteoarthritis of the hip is among the most common diseases affecting the musculoskeletal system with a prevalence of approximately 8% in the general adult population [1]. Surgical treatment needs to be considered in the case of non-responsiveness to conservative treatment. Total hip arthroplasty is a common procedure for end stage osteoarthritis with good results reported in the literature [2]. There are multiple surgical approaches that can be used, including the transgluteal (lateral), posterior, and antero-lateral approach [3]. In 2004, the minimally-invasive total hip arthroplasty via the direct anterior approach has been reported as a viable surgical technique. This approach, using the anterior intermuscular and internervous interval, was initially described by Carl Hueter in 1881 [4, 5]. Anterior Minimally Invasive Surgery (AMIS) is frequently performed with a traction table, and has been reported as a safe and reliable surgical technique [3, 6–8].

**Fig. 1:**
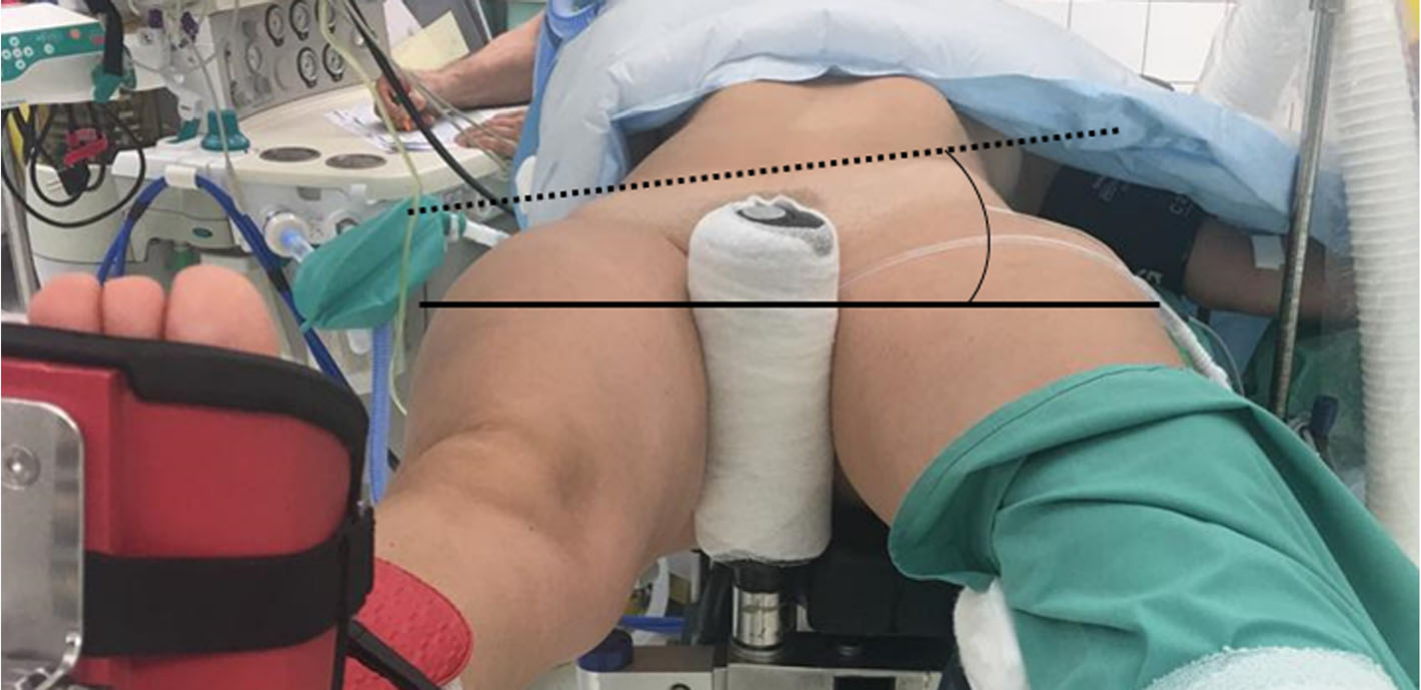
A sample illustration of aPT towards the side of operation when positioned on the traction table. The curved line represents the aPT (in this case represented as a negative value), measured between the horizontal plane (bold line) and the line connecting both ASIS (dotted line)

However, based on observations of the authors, the positioning of the patient on the traction table may result in an axial pelvic tilt (aPT) of the patient’s pelvis towards the side of operation, leading to the operative side coming to lie lower than the non-operative side (Fig. 1). In recent years, the role of sagittal pelvic tilt in THA has been evaluated in multiple studies [9–12]. While prior investigations have demonstrated a significant influence of patient position on sagittal pelvic tilt [9], the influence of traction table utilization on aPT during direct anterior THA remains unknown.

The aim of the present study was to assess aPT in supine positioning of the patient with and without the utilization of a traction table, during direct anterior THA. Furthermore, we investigated potential preoperative risk factors for an aPT of more than 5 degrees.

## Methods

### Study population

The present study was approved by the Institutional Review Board (IRB). Patients were included who underwent THA via the *Anterior Minimally Invasive Surgery* (AMIS) technique using a traction table (*Medacta International SA, Castel San Pietro, Switzerland*) between 11/2018 and 03/2019 at a single institution. During the study period, a total number of 390 patients underwent DAA THA, with 71 cases performed by the principal investigator. Exclusion criteria were defined as unavailable data, an age at surgery < 18 years, revision total hip arthroplasty, unavailable preoperative standing antero-posterior plain radiographs of the pelvis with calibration markers, as well as a refusal to participate in research studies.

### Data collection

**Fig. 2:**
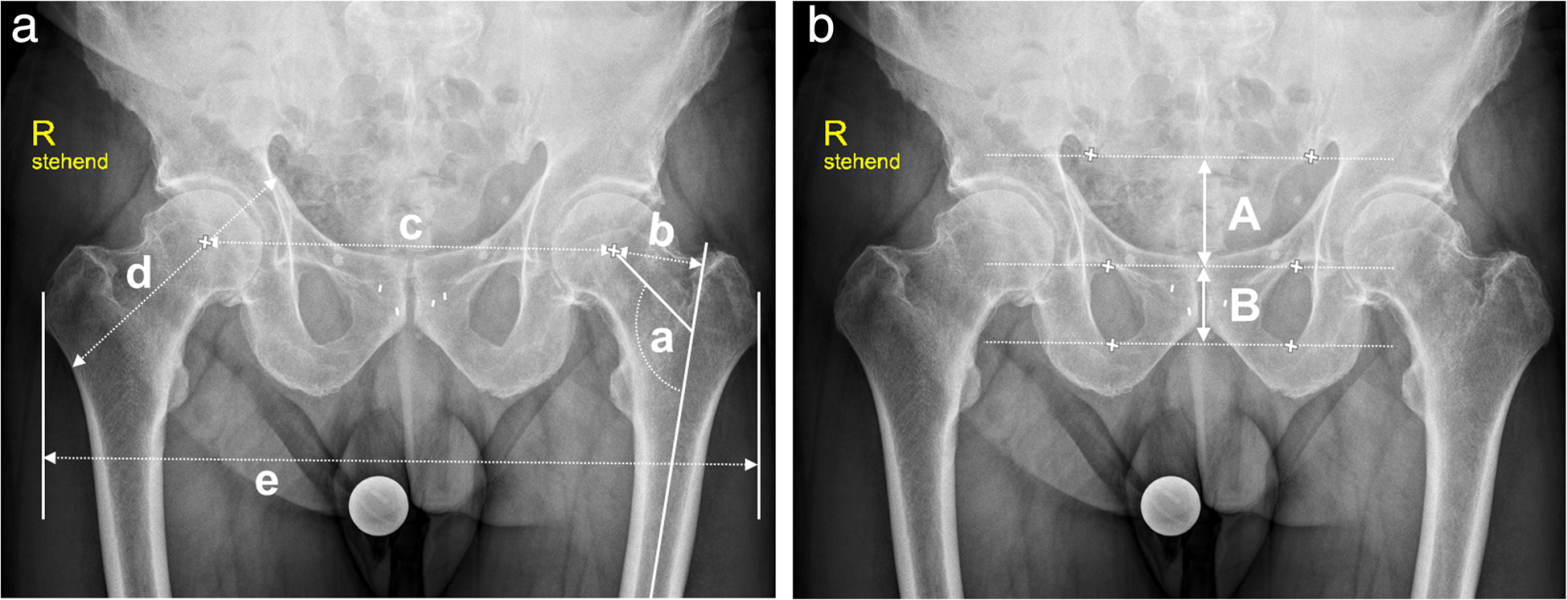
**a** [a] caput-collum-diaphyseal angle, [b] femoral offset, [c] distance between the centers of the femoral heads of both hips, [d] hip axis length, and [e] intertrochanteric distance. **b**. Pelvic tilt ratio (B/A) for sagittal pelvic tilt estimation according to Schwarz et al. [14]

Data were collected on age, gender, height, weight, body mass index (BMI), and several preoperative radiographic parameters. Additionally, data were gathered on the patient’s preoperative pelvic position (a) in the supine position on the operating table, and (b) after positioning on the traction table with the foot mounted in the foot boot. Preoperative standing antero-posterior pelvic radiopraphs were analyzed for the caput-collum-diaphyseal (CCD) angle, the femoral offset, the distance between the centers of both femoral heads, the distance between the most lateral edges of both greater trochanters, the hip axis length [13], the tilt ratio calculation and sagittal pelvic tilt estimation according to Schwarz et al. [14] The CCD angle is defined as the femoral neck-shaft angle, and was measured between the longitudinal axes of the femoral neck and the femoral shaft. The femoral offset was measured as the perpendicular distance between the longitudinal femoral shaft axis and the center of the femoral head. Furthermore, an offset ratio was calculated by dividing the femoral offset by the distance between the centers of both femoral heads. All plain radiographic measurements were performed after calibration using a preoperative planning software (*mediCAD® Classic v4.5, mediCAD Hectec Gmbh, Germany*). (Fig. 2).

### Measurement of pelvic position

**Fig. 3:**
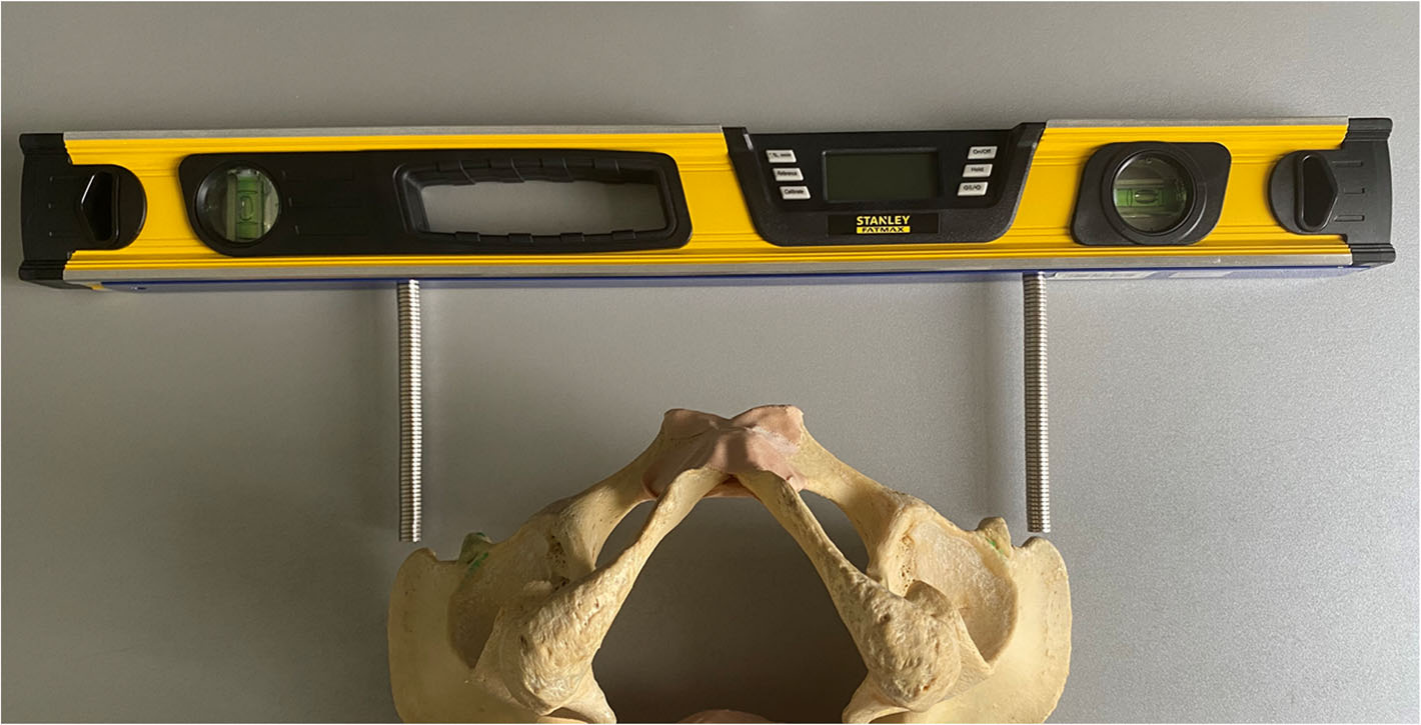
Axial sample illustration of palpation technique of ASIS using the digital inclinometer

The bilateral anterior superior iliac spines (ASIS) were defined as the reference for measuring the axial pelvic orientation relative to the horizontal plane. (Fig. 1) A digital inclinometer (*Stanley Black & Decker, New Britain, CT*) was routinely used to assess (a) the pelvic position on the regular operating table in the supine position, and (b) the pelvic position after the leg has been mounted in the foot boot of the traction table, with the difference then calculated. The patient was positioned on the traction table, with the table in neutral position (no traction, no abduction, no adduction), with the foot boot fixed at the height of the patient’s pelvis. Two identical magnetic pedestals were used for accurate palpation of the ASIS, as illustrated in Fig. 3. Each measurement was performed twice with the average calculated. An aPT towards the side of operation (operated hip joint lower relative to the contralateral side) is reported with a negative value, whereas an aPT towards the contralateral hip joint (operated hip joint higher relative to the contralateral side) is reported with a positive value. In case of aPT, the patient’s position is preoperatively corrected by the surgeon to a neutral position. All measurements were performed by the same consultant surgeon as a pilot project as opposed to an institutional standard (non-consecutive patient inclusion).

### Statistical analysis

Categorical variables are reported as frequencies and percentages, whereas continuous variables are reported as mean ± standard deviation (SD) for parametric data and as median with interquartile range (IQR) for non-parametric data. The Pearson’s chi squared test, or Fisher’s exact test if the expected cell count in any cell was < 5, were used for the comparison of proportions between groups. In order to test for a normal distribution of data, the Kolmogorov Smirnov test was used. In case of a parametric distribution of continuous data, the Student’s t-test was applied (unpaired/paired), whereas the Mann-Whitney U test (unpaired) was used for the comparison of continuous variables in case of a non-parametric distribution. A *p*-value of < 0.05 was considered statistically significant. Data analysis was performed using IBM SPSS Statistics, Version 23.0 (*IBM Corp., Armonk, NY*).

## Results

### Study population

During the study period a total of 50 patients (F: 32; M: 18) met the previously defined inclusion criteria. The average age at surgery was 60.6 ± 13.6 (range: 26.5 to 88.3) years, and the average BMI was 27.2 ± 5.0 (range: 17.9 to 41.5) kg/m^2^.

### Measurement of pelvic position

**Fig. 4:**
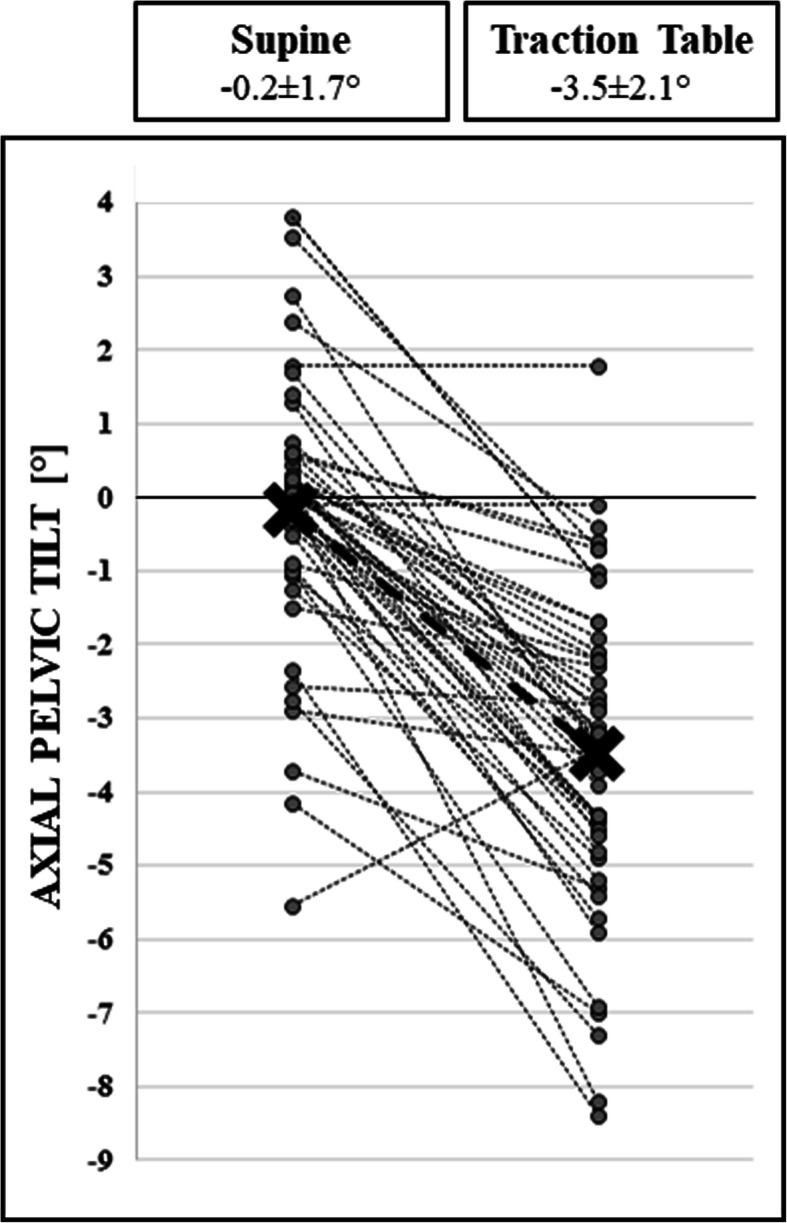
The left and right columns indicate values for aPT before, and after the leg has been mounted on the traction table device, with average values of -0.2 ± 1.7° and -3.5 ± 2.1°, respectively. The bold line indicates the average change of aPT within the entire study population (*n* = 50) from the supine position to the position on the traction table (*p* < 0.001)

There were 47 patients (94.0%) with downward aPT towards the side of operation, 2 patients (4.0%) with no change of pelvic position, and 1 patient (2.0%) with upward aPT, when comparing the measurements (a) in supine position, and (b) after the leg has been mounted in the foot boot of the traction table, respectively. The aPT in supine position (before positioning on the traction table) was − 0.2 ± 1.7 (range: − 5.6 to 3.8) degrees. After positioning on the traction table, the aPT was measured as − 3.5 ± 2.1 (− 8.5 to 1.6) degrees (*p* < 0.001). (Fig. 4).

There were 21 patients (53.9%) with an absolute aPT towards the operated side of more than 4 degrees, and 9 patients (23.1%) with an absolute aPT towards the operated side of more than 5 degrees.

### Radiographic analysis

The average preoperative values were calculated for the CCD angle as 132.4 ± 8.7 (range: 109.5 to 146.7) degrees, the femoral offset as 37.4 ± 6.7 (range: 17 to 51) mm, the distance between the centers of the femoral heads as 184.4 ± 13.7 (range: 137 to 208) mm, the distance between the most prominent edges of both greater trochanters as 312.9 ± 17.7 (range: 275 to 350) mm, and the hip axis length as 115.6 ± 14.7 (range: 79 to 148) mm. The offset ratio was calculated as a median of 0.20 (IQR: 0.04). Based on the sagittal pelvic tilt calculation formula published by Schwarz et al. [14], the mean sagittal pelvic tilt was estimated as − 0.8 ± 5.7 (range: − 11.6 to 15.9) degrees on preoperative X-rays. (Table 1).

**Table 1:** Demographic variables of the study population

	Variables
**Female gender**	64.0%
**Age (years)**	60.6 ± 13.6 (range: 26.5–88.3)
**BMI (kg/m**^**2**^**)**	27.2 ± 5.0 (range: 17.9–41.5)
**CCD angle (degrees)**	132.4 ± 8.7 (range: 109.5–146.7)
**Femoral offset (mm)**	37.4 ± 6.7 (range: 17.0–51.0)
**Distance between femoral head centers (mm)**	184.4 ± 13.7 (range: 137.0–208.0)
**Distance between greater trochanters (mm)**	312.9 ± 17.7 (range: 275.0–350.0)
**Hip axis length (mm)**	115.6 ± 14.7 (range: 79.0–148.0)
**Sagittal pelvic tilt (degrees)**	−0.8 ± 5.7 (range: − 11.6-15.9)
**Offset ratio**	0.20 (IQR: 0.04)

### Sub-analysis: comparison of patients with more or less than 5° of aPT

The cut-off value of > 5° was defined based on the overall average difference of pelvic position of − 3.3 with 1 standard deviation of − 2.0 degrees. Those patients who had an aPT greater than 5 degrees after positioning on the traction table, had a significantly lower CCD angle than those patients who had an aPT less than 5 degrees (125.5 ± 10.7 degrees compared to 133.9 ± 7.6 degrees on average, *p* = 0.007).

There were no statistically significant differences between both groups with regard to age, gender, BMI, femoral offset, distance between the centers of both femoral heads, distance between the most lateral edges of both greater trochanters, hip axis length, sagittal pelvic tilt, and offset ratio. Details of the univariate statistical comparison are listed in Table 2.

**Table 2:** Comparison of patients with vs. without an aPT of more than 5 degrees

	aPT > 5° (***n*** = 9)	aPT < 5° (***n*** = 41)	***p***-Value
**Female gender**	88.9%	58.5%	0.130
**Age (years)**	56.4 ± 13.9 (range: 40.9–74.5)	61.5 ± 13.5 (range: 26.5–88.3)	0.307
**BMI (kg/m**^**2**^**)**	26.7 ± 4.6 (range: 21.9–37.0)	27.4 ± 5.1 (range: 17.9–41.5)	0.735
**CCD angle (degrees)**	125.5 ± 10.7 (range: 109.5–141.7)	133.9 ± 7.6 (range: 116.3–146.7)	**0.007**
**Femoral offset (mm)**	39.2 ± 10.0 (range: 17–51)	37.0 ± 5.8 (range: 20–49)	0.360
**Distance between femoral head centers (mm)**	186.6 ± 10.6 (range: 170–204)	184.0 ± 14.4 (range: 137–208)	0.611
**Distance between greater trochanters (mm)**	311.3 ± 18.8 (range: 281–335)	313.2 ± 17.7 (range: 275–350)	0.779
**Hip axis length (mm)**	108.1 ± 17.2 (range: 82–133)	117.2 ± 13.8 (range: 79–148)	0.094
**Sagittal pelvic tilt (degrees)**	−0.8 ± 5.0 (range: −7.1-6.7)	−0.8 ± 5.9 (range: −11.6-15.9)	0.999
**Offset ratio**	0.22 (IQR: 0.07)	0.20 (IQR: 0.04)	0.220

## Discussion

The aim of the present study was to evaluate the patient’s pelvic position in the axial plane when performing THA via the AMIS technique using a traction table. This study shows that positioning of the patient on the traction table during THA via the direct anterior approach frequently results in an aPT towards the operative side. In fact, 94.0% of patients showed aPT towards the side of operation. On average, the patient’s pelvis axially tilted towards the side of operation by 3.3 ± 1.9 degrees. A decreased CCD angle was associated with an aPT of more than 5 degrees, which might be explained by changes in the lever arm acting on the hip joint. The average femoral offset was minimally higher in patients with an aPT of more than 5 degrees. Furthermore, 88.9% of patients with an aPT of more than 5 degrees were women, with only 58.5% women in the subgroup of patients with an aPT of less than 5 degrees. However, non-significant findings may be related to the small sample size of the present study, potentially resulting in a type II error. Further studies with significantly larger sample sizes are required to reduce the risk of type II error when assessing the relationship between these variables and aPT.

In recent years, there has been great interest in the effects of sagittal pelvic tilt on THA [9–12]. In fact, Kalteis et al. reported a correlation between sagittal pelvic tilt and cup position, with greater tilt associated with greater inclination and anteversion of the cup [10]. While sagittal tilt may therefore influence cup position, the effects of aPT on cup position are unknown. Additionally, we did not note any significant relationship between pre-operative sagittal pelvic tilt and traction table aPT. Sagittal pelvic tilt could not be radiologically assessed on the traction table since fluoroscopic images obtained intra-operatively did not include the whole of the pelvis.

While there are several investigations on influence of sagittal pelvic tilt on acetabular anteversion [11] [12], there is no study evaluating the influence of aPT (rotation) on real anteversion during THA via the AMIS technique. Although the present study cannot assess the influence of aPT on real cup anteversion, to the authors’ knowledge it is the first of its kind to assess axial pelvic malrotation on the traction table during THA via the AMIS technique. This may have potential, yet unknown, influence on acetabular cup position. However, navigated total hip arthroplasty would correct for this alteration in axial pelvic tilt.

There are limitations that need to be considered when interpreting the reported findings. One of the main limitations of the study is that the potential association between aPT and the postoperative acetabular cup version could not be evaluated. This is due to the fact that each the surgeon corrected the patient’s position to a neutral position if an aPT was noticed on the traction table. Another limitation is the fact that boot height was not measured as a separate value, potentially influencing aPT. Furthermore, this study was a non-consecutive series with a small sample size and therefore may be prone to selection bias or type II error. Additionally, extrapolation of results to other surgeons’ practice may not be appropriate. Finally, measurements were performed on two-dimensional plain radiographs, rather than on three-dimensional imaging modalities such as computed tomography or magnetic resonance imaging. This may lead to concerns that measurements made on 2-D imaging techniques are unable to account for non-planar or rotational anatomical features (e.g. femoral anteversion). However, others have reported good validity and reliability for assessing proximal femoral geometry on internal rotation radiographs of the hips [15]. Nevertheless, this fact needs to be considered when interpreting the results of radiographic assessment. Based on the presented results, we do not recommend to globally use a digital inclinometer for all supine THA surgeries using the AMIS method, however, we want to underline the importance of evaluating patient position on the traction table before initiating surgery.

## Conclusions

This study confirms the frequent occurrence of aPT towards the side of operation during THA via the AMIS technique using a traction table, with unknown clinical relevance. Especially surgeons on the early phase of their learning curve should raise their awareness for this potential obstacle during patient positioning.

## Data Availability

All data generated or analysed during this study are included in this published article and its supplementary information files.
